# Supplementary material for: Content-rich biological network constructed by mining PubMed abstracts
Source: BMC Bioinformatics. 2004 Oct 8;5:147. doi: 10.1186/1471-2105-5-147 (PMC528731; doi:10.1186/1471-2105-5-147)
Supplement: Additional File 2 — The original results of the above study (non-essential files are deleted to keep the file size under the limit set by BMC bioinformatics). [file 1471-2105-5-147-S2.bz2 › chilibotAdditionalFile2/dip05/721ID11799122E18058/html/ENVZ_OMPR.html]

 


 **ENVZ** and **OMPR** 
  
Found 178 abstracts in PubMed, retrieved 05.  
 

 What does Google say? 
 PDF only 
| .edu only 

---

**Interactive relationship** (e.g. stimulation, inhibition, etc)

**Non-interactive relationship** (e.g. studied together, co-existance, homology, etc.)

- **EnvZ**  and  **OmpR**  are a transmembrane sensor and its cognate response regulator, respectively, regulating the transcription of porin genes in response to medium osmolarity in Escherichia coli.  Ref: 12754242 J Bacteriol, 2003
- The operon flhDC is controlled by numerous regulators including H NS, CRP,  **EnvZ**   **OmpR** , QseBC and LrhA.  Ref: 12864862 Mol Microbiol, 2003
- The  **EnvZ**   **OmpR**  histidyl aspartyl phosphorelay HAP system in Escherichia coli regulates the expression of ompF and ompC, the major outer membrane porin genes, in response to environmental osmolarity changes.  Ref: 12767831 J Mol Biol, 2003
- In alpha proteobacteria, HPRK is located downstream of genes encoding a two component system of the  **EnvZ**   **OmpR**  family.  Ref: 12867744 J Mol Microbiol Biotechnol, 2003
- The phosphoryl group of H6 HepK is transferred rapidly and efficiently to both H6DevR and H6DevRA but not to His tagged  **OmpR** , whose cognate sensor kinase is  **EnvZ** .  Ref: 12637541 J Biol Chem, 2003
